# Leaf hydraulic conductance is linked to leaf symmetry in bifacial, amphistomatic leaves of sunflower

**DOI:** 10.1093/jxb/eraa035

**Published:** 2020-01-23

**Authors:** Freya Richardson, Gregory J Jordan, Timothy J Brodribb

**Affiliations:** 1 School of Natural Sciences, University of Tasmania, Hobart, Tasmania, Australia; 2 University of Cambridge, UK

**Keywords:** Amphistomaty, hydraulic conductance, *K*_leaf_, leaf water potential, mesophyll, stomata, stomatal ratio, vein density

## Abstract

The hydraulic implications of stomatal positioning across leaf surfaces and the impact on internal water flow through amphistomatic leaves are not currently well understood. Amphistomaty potentially provides hydraulic efficiencies if the majority of hydraulic resistance in the leaf exists outside the xylem in the mesophyll. Such a scenario would mean that the same xylem network could equally supply a hypostomatic or amphistomatic leaf. Here we examine leaves of *Helianthus annuus* to determine whether amphistomaty in this species is associated with higher hydraulic efficiency compared with hypostomatic leaves. We identified asymmetry in the positioning of minor veins which were significantly closer to the abaxial than the adaxial leaf surface, combined with lower *K*_leaf_ when transpiration was driven through the adaxial rather than the abaxial surface. We also identified a degree of coordination in stomatal behaviour driven by leaf hydraulics, where the hydraulic conditions experienced by an individual leaf surface affected the stomatal behaviour on the opposite surface. We found no advantage to amphistomaty based on efficiencies in construction costs of the venous system, represented by vein density:stomatal density, only limited hydraulic independence between leaf surfaces. These results suggest that amphistomaty does not substantially increase whole-leaf hydraulic efficiency.

## Teaser

Anatomical asymmetries between the leaf surfaces lead to different hydraulic conductances when transpiration is driven through each surface. These results provide physiological evidence of potential trade-offs associated with amphistomaty.

## Introduction

The distribution of stomata, the microscopic pores through which plants take up carbon dioxide (CO_2_) and consequently lose water, is a key functional characteristic of leaves. Leaves with stomata restricted to the lower surface are described as ‘hypostomatic’, and leaves with stomata distributed across both surfaces are ‘amphistomatic’, although the number of stomata on one surface may be greater than that on the other. At a global scale, both hypostomatic and amphistomatic leaves are common; however, leaves with stomata restricted to the upper surface (‘hyper’- or ‘epistomatic’) are relatively uncommon. While stomatal distributions between leaf surfaces have been correlated with a number of environmental factors, the exact functional implications with regard to costs and benefits of amphistomaty remain unclear but are important in understanding broad evolutionary patterns (Drake *et al*., 2019).

Amphistomaty has been correlated with both high light environments (Mott and Michaelson, 1991; Jordan *et al.*, 2014) and fast growth or herbaceousness (Muir, 2015, 2018). Suggested advantages to amphistomaty include increased CO_2_ supply to the mesophyll (Parkhurst, 1978; Beerling and Kelly, 1996) associated with both the additional epidermal space allocated to stomata for amphistomatic leaves (Muir, 2018) and the reduction of the mesophyll pathway length for CO_2_ following uptake via the stomata to the site of photosynthesis (Parkhurst, 1978, 1994). Possible explanations of why the amphistomatic leaf form is not more common globally include increased susceptibility to pathogen infection (McKown *et al.*, 2014), lower water use efficiency associated with a temperature gradient between the adaxial and abaxial leaf surfaces (Richardson *et al.*, 2017), greater investment in light harvesting, the potential for upper stomata to be blocked by water droplets, and inefficient leaf structure in light-limited rather than carbon-limited environments (Muir, 2015).

Hydraulic efficiency may also limit the benefits of amphistomaty and, despite consideration of the hydraulic implications of stomatal positioning on the leaf in previous studies (Mott, 2007; Buckley *et al.*, 2017; Richardson *et al.*, 2017), potential hydraulic advantages or disadvantages associated with amphistomaty are not well understood. Pathways for water movement within the leaf can constitute >30% of the total hydraulic resistance for the entire plant (Sack and Holbrook, 2006). Leaf hydraulic limitations can place significant constraints on plant functional processes, which is reflected in strong correlations between leaf hydraulic conductance (*K*_leaf_), stomatal conductance to water vapour (*g*_s_), and maximum photosynthetic assimilation (Brodribb *et al.*, 2007; Damour *et al.*, 2010). The ability to simultaneously supply water to both leaf surfaces via a single vascular network offers amphistomatic leaves a potential advantage in minimizing building costs. To balance the investment in the water supply network with the potential transpirational demand created by the stomata, an increase in *g*_s_ in amphistomatic leaves should also demand an increased capacity for the vascular network to supply water. Previous work suggests complete independence in the operation of stomata on the two surfaces of some amphistomatic leaves in response to light and CO_2_ (Mott and Peak, 2018), as well as evaporative demand (Mott and Parkhurst, 1991; Richardson *et al.*, 2017). Hydraulic independence of leaf surfaces despite their reliance on a common supply network would make amphistomaty highly advantageous by allowing amphistomatic leaves to overcome apparently universal constraints on the number of stomata per unit vein length (Carins Murphy *et al.*, 2014). Any advantage to amphistomaty based on an increase in stomatal density (*S*_D_) must depend on the amount of time stomata on both surfaces are able to open compared with the extra costs of increasing both *S*_D_ and vein density (*V*_D_).

Additionally, the general anatomical form of the leaf may affect leaf hydraulics. Amphistomatic leaves are not morphologically uniform; variation in the ratio of stomata on the adaxial surface to those of the total leaf (*S*_R_) can occur both within and between species. Additionally, amphistomatic leaves can be isobilateral, where the abaxial and adaxial tissues are symmetrical, or bifacial, where the mesophyll cells within the upper and lower portions of the leaf are differentiated into palisade and spongy mesophyll tissue but not necessarily evenly so. The asymmetry of the bifacial leaf is likely to cause asymmetrical hydraulic conductances to different surfaces if the hydraulic pathways from the veins to the adaxial and abaxial stomata vary in length and/or cell types that have different resistances. The dominant hydraulic pathway can be dynamic and is determined by whether an amphistomatic leaf is transpiring through both surfaces (i.e. the leaf is functionally amphistomatic), the lower surface only (functionally hypostomatic), or the upper surface only (functionally epistomatic). Buckley *et al.* (2015) modelled the movement of water vapour outside the xylem, concluding that transpiration occurring through both surfaces changes the vertical gradient in water potential within the leaf such that tissues above and below the xylem are both connected to the transpiration stream, and predicting that the lowest water potential should occur in the mesophyll cells close to the epidermis (Buckley *et al.*, 2015).

The documented independent stomatal closure both between surfaces (Mott, 2007; Richardson *et al.*, 2017; Mott and Peak, 2018) and laterally across a surface through ‘patchy’ stomatal closure (Mott *et al.*, 1993) suggests that stomatal closure driven by evaporative demand is due to responses to localized water potential or cell turgidity gradients. Recent research suggests that the site of foliar abscisic acid (ABA) biosynthesis is primarily within the leaf mesophyll tissue and is synthesized in response to water potential and associated cell turgor loss (McAdam and Brodribb, 2016, 2018). This localized synthesis may explain the independent stomatal closure on the leaf surfaces when different surfaces are exposed to different evaporative conditions. However, we would still expect to see some connectivity in the stomatal behaviour between the two surfaces when bulk leaf water potential is affected.

Previous research into the independent stomatal response between leaf surfaces of amphistomatic leaves has done so by increasing the evaporative demand on one surface and monitoring subsequent changes in gas exchange for both surfaces (Mott, 2007; Mott and Peak, 2018). These results suggest strong independence of stomatal movement between surfaces, concluding that stomata were responding only to pressure gradients generated downstream of the vascular tissue (Mott, 2007); this is consistent with the idea that the greatest hydraulic resistance and therefore the greatest gradient in water potential within the leaf occurs outside the xylem (Cochard *et al.*, 2004; Brodribb *et al.*, 2007; Buckley *et al.*, 2015). Alternatively, under a scenario where greater pressure gradients exist within the xylem (Sack *et al.*, 2004; Zwieniecki *et al.*, 2007), changes in bulk leaf water potential which could be driven by increased evaporative demand and higher transpiration rates on one surface should impact the gas exchange for the entire leaf, including the opposite surface. The effects on gas exchange for both leaf surfaces would be likely to be strongest when a leaf is hydraulically limited or operating at a high *g*_s_.

Here we measured hydraulic properties of different leaf surfaces in amphistomatic leaves in an attempt to reconcile stomatal behaviour with hydraulic anatomy. Our overall aims were to: (i) test whether morphological abaxial/adaxial leaf asymmetry corresponds to differences in hydraulic conductance; and (ii) determine whether stomatal behaviour between surfaces is connected. We used a herbaceous species (*Helianthus annuus*) as an example of a common amphistomatic species with strong bifacial differentiation. We therefore hypothesized that this species should exhibit asymmetrical hydraulic conductances between leaf surfaces leading to different water potential gradients associated with the two leaf surfaces and preferential closure of stomata on the surface where the pathway has greatest hydraulic resistance under high evaporative demand. Additionally, we hypothesized that stomata on the two leaf surfaces should be responsive to changing conditions on the opposite leaf surface via changes in leaf water balance that are driven by transpiration.

## Materials and methods

### Plant material

*Helianthus annuus* L. var. *sunfola* plants were grown in the glasshouse facility at the University of Tasmania Sandy Bay campus. Plants were grown in 3 litre pots under controlled conditions and kept well watered; variation in initial leaf water potential was considered when analysing the transpiration and leaf hydraulic conductance data.

### Leaf anatomy

The *S*_D_ of two leaves from each of four plants of *H. annuus* was determined by bleaching small sections of leaf tissue (~2 cm^2^) in household bleach until clear, and staining with toluidine blue (Carins Murphy *et al.*, 2012); epidermes were not removed and the cuticle remained intact. Sections were mounted on glass slides in phenol glycerine jelly and photographed using a Nikon DS Fi2 camera (Melville, NY, USA) mounted on a Leica DM 1000 microscope (Nussloch, Germany). Stomata were counted across five fields of view (FOV) at ×20 magnification (FOV 0.141 mm^2^) per surface of each leaf from the photographs using ImageJ software (National Institutes of Health, Bethesda, MD, USA). The stomatal ratio (*S*_R_) was calculated as *S*_D adaxial_/*S*_D total_, such that an *S*_R_ of 0.5 indicates an evenly amphistomatic leaf, an *S*_R_ of 0 indicates a fully hypostomatic leaf, and an *S*_R_ of 1 indicates a fully epistomatic leaf.

Vein density (*V*_D_) was measured from the same bleached leaf sections used for stomatal counts. The total length of leaf veins was measured for three images from each section taken at ×4 magnification (FOV 3.471 mm^2^) using ImageJ software to trace and measure the length of the minor veins. Mean values of both *S*_D_ and *V*_D_ for each leaf were used for analysis.

Leaf thickness and the minimum distances from the minor vein to the adaxial and abaxial surfaces were measured on leaf cross-sections. Cross-sections were prepared as follows. Tissue from one leaf from each of three individual *H. annuus* plants was fixed overnight in 4% paraformaldehyde with phosphate-buffered saline with gentle agitation. The samples were then dehydrated at room temperature for 45 min in 12, 25, 50, 75, 95, and 100% ethanol and embedded in polyethylene glycol (PEG) 1000. Once the samples were set, cross-sections were cut using a rotary microtome (HM340 E, Microm International, Walldorf, Germany) and mounted on glass slides in phenol glycerine jelly. Leaf cross-sections were photographed at ×20 magnification (FOV 0.141 mm^2^) and the images were used to measure leaf thickness (*T*_leaf_) and the distance from the minor veins to the inside of the adaxial and abaxial epidermis (*D*_upper_ and *D*_lower_). Four measurements per leaf were made for *T*_*l*eaf_, *D*_upper_, and *D*_lower_.

### Leaf hydraulic conductance

Leaf hydraulic conductance was measured during the middle of the day between 10.00 h and 13.00 h using the evaporative flux method (Sack *et al.*, 2002; Brodribb and Holbrook, 2006). The projected leaf area of the leaves used varied to a minor extent but all leaves were between 0.003 m^2^ and 0.01 m^2^. Prior to each measurement, an initial leaf water potential (Ψ _leaf_) was obtained by measuring a neighbouring leaf using a Scholander pressure chamber. The target leaves were cut through the petiole whilst submerged underwater and immediately connected via the petiole to a flow meter (with zero upstream pressure gradient). The leaf was then removed from the water and gently dried. To reproduce the natural leaf orientation of the species, leaves were orientated horizontally with 600 µmol m^–2^ s^–1^ light applied only to the adaxial surface. To encourage transpiration, leaves were heated evenly on both leaf surfaces by a stream of warm air (between 24 °C and 30 °C). Transpirational flux was recorded once a steady state had been reached (<10% variation over 180 s), and leaves were subsequently disconnected from the flow meter, immediately wrapped in damp paper towel, and a final Ψ _leaf_ was measured using the pressure chamber. Wrapped leaves were then left to equilibrate within sealed plastic bags and Ψ _leaf_ was re-measured using the pressure chamber after 30 min, and again after each 30 min interval until results were stable over two consecutive measurements (up to 90 min after disconnection from the flow meter). *K*_leaf_ was calculated as:

$$\begin{array}{l} K_{leaf}=F/\Psi_{leaf}\; \end{array}$$

Where *K*_leaf_ is the hydraulic conductance, *F* is the transpirational flux, and Ψ _leaf_ is the water potential at steady state. Leaf hydraulic conductance values were standardized for projected leaf area and for the viscosity of water at 20 °C, using an empirical function based on data from Korson *et al.* (1969).

To ensure that laboratory conditions were sufficient to open stomata simultaneously on both surfaces of amphistomatic (unmodified) leaves, we tested whether amphistomatic, hypostomatic, and epistomatic leaves showed different transpiration rates (*E*), assuming that higher *E* in amphistomatic leaves than in epistomatic or hypostomatic leaves would indicate water loss from both surfaces of the leaf.

In 14 leaves the hydraulic conductance of a ‘hypostomatic’ configuration was investigated by covering the adaxial leaf surface with clear plastic adhesive tape, such that transpirational water loss was restricted to the uncovered, abaxial surface. In 14 leaves an ‘epistomatic’ configuration was simulated by applying the tape to the abaxial surface rather than the adaxial surface. The tape covers were applied to the leaves in the morning at ~09.00 h on the day that measurements were taken, with leaves equilibrating for a minimum of 1 h prior to the first measurement.

### Gas exchange

We examined the independence of stomata between abaxial and adaxial leaf surfaces by modifying the evaporative demand on one leaf surface independent of the other using a portable infrared gas analyser (IRGA; GFS-3000, Heinz Walz, Effeltrich, Germany) with a standard measuring head 3010-S and LED array.

**Fig. 1.**
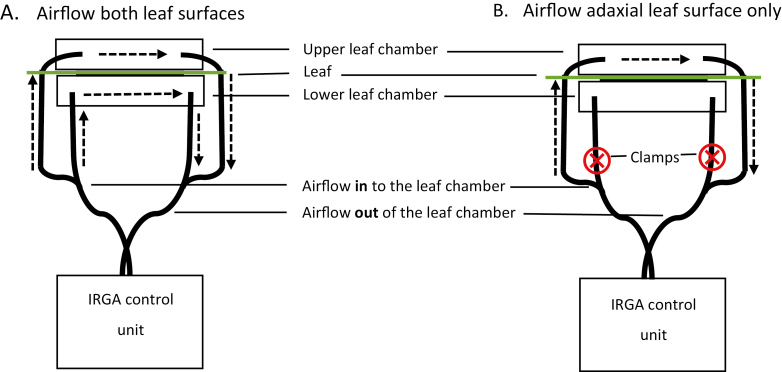
Schematic diagram of airflow between the WALZ GFS-300 control unit and the standard measuring head. Arrows show the direction of airflow. (A) The airflow when both leaf surfaces are measured. (B) The airflow when measuring the adaxial leaf surface only. The symbol ‘⨂’ shows the location where compression clamps were applied to re-route all airflow through the upper portion of the leaf chamber when required. (This figure is available in colour at *JXB* online.)

Five leaves from three well-watered *H. annuus* plants were enclosed one at a time within the cuvette with each individual leaf completely filling the chamber surface with a projected leaf area of 8 cm^2^. Chamber conditions were maintained at 25 °C with ambient CO_2_, and light intensity of 1500 µmol m^–2^ s^–1^. Leaves were allowed to reach a stable *g*_s_ under low vapour pressure difference (VPD) conditions (<1.2 kPa) while gas exchange parameters were logged every 20 s. After *g*_s_ had stabilized, compression clamps were applied to the air inflow tube supplying the lower leaf chamber and the air outflow tube from the lower leaf chamber, as shown in Fig. 1. Mixing fans were assumed to be effective in maintaining the boundary layer. Mixing fans were set to a maximum rate to minimize changes to boundary layer conditions when airflow was diverted to a single surface. This allowed an instantaneous gas exchange measurement of the adaxial leaf surface to be made once values had stabilized (~60 s), after which clamps were removed from airflow tubes. The VPD was then increased. Leaves were then allowed to stabilize under high VPD (>2.2 kPa) (~20 min). At this point, the response of adaxial stomata to a steep reduction in transpiration from the lower surface was examined by blocking flow to the lower leaf chamber. To do this, the air flow to the lower leaf surface was blocked, allowing the lower leaf chamber to humidify, reducing transpiration from the abaxial leaf surface. VPD was maintained in the upper leaf chamber for the adaxial leaf surface, and gas exchange was logged to establish whether the reduction in abaxial transpiration resulted in an increase in adaxial *g*_s_.

The difference between the relative humidity in the chamber and fully saturated air (i.e. 1–relative humidity) for each second of time after isolation of the abaxial leaf surface was estimated using an exponential decay function, based on the initial values for transpiration rate and relative humidity of the air, as well as the volume of the chamber. The time to 95% saturation with water vapour was calculated by solving this function for time. This calculation assumes that abaxial *g*_s_ and the temperature of the chamber remained constant. If the abaxial stomata opened (i.e. abaxial *g*_s_ increased), the chamber would reach 95% saturation more quickly.

### Data analysis

Paired *t*-tests were used to compare *S*_D abaxial_ and *S*_D adaxial_, and *D*_abaxial_ and *D*_adaxial_. A linear regression model was fitted to a pairwise scatter plot to analyse the relationship between *V*_D_ and √*S*_D_. ANOVAs were used to compare the transpiration rates between transpiring leaf surfaces during hydraulic measurements with final and initial Ψ _leaf_ included as factors, as well as *K*_leaf_ of functionally hypostomatic and functionally epistomatic *H.annuus* leaves, with *E*, and final and initial Ψ _leaf_ included as factors.

Paired *t*-tests were used to compare *g*_s adaxial_ and *C*_i adaxial_ measurements for *H. annuus* leaves prior to and following a VPD transition, and prior to and following cessation of abaxial water loss.

Analyses were undertaken in R (R Core Team, 2014).

## Results

### Leaf description

**Fig. 2.**
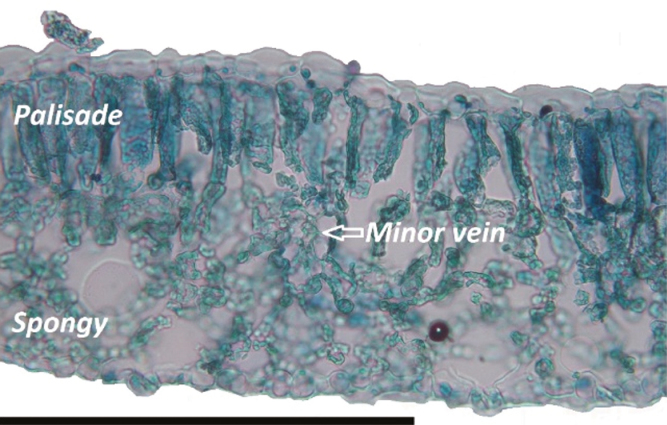
Example leaf cross-section *H. annuus* with the adaxial leaf surface at the top and the abaxial below. Palisade and spongy mesophyll cells are clearly segregated. Scale bar=0.2 mm. (This figure is available in colour at *JXB* online.)

*Helianthus annuus* leaves grown under the conditions described above for these experiments were amphistomatic, with a mean *S*_R_ of 0.41 (±0.03). The *S*_D abaxial_ [126.81 (±17.3) mm^–2^ leaf area] was significantly higher than the *S*_D adaxial_ [89.44 (±13.2) mm^–2^ leaf area] (Table 1). Mesophyll tissue within *H. annuus* leaves was clearly segregated into palisade mesophyll beneath the adaxial epidermis and spongy mesophyll above the abaxial leaf surface (Fig. 2). On average, the abaxial leaf surface was significantly closer to minor veins than the adaxial surface (Table 1).

**Table 1.** Leaf description and anatomical measurements (mean ±SD)

*Leaf trait*	
**Leaf description**	Herbaceous, horizontal, bifacial leaves
**Leaf thickness (*T***_**leaf**_)	0.19±0.02 mm
Distance from vein to epidermis (*D*)	
Abaxial	0.05±0.01 mm
Adaxial	0.07±0.01 mm
Difference between surfaces	*t*= –5.25; *P*<0.0001***
***Stomatal density* (*S***_**D**_)	
Total	216.3±27.6 mm^–2^ leaf area
Abaxial	126.8±17.3 mm^–2^ leaf area
Adaxial	89.4±13.2 mm^–2^ leaf area
Difference between surfaces	*t*=7.69; *P=*0.0001***
**Stomatal ratio (*S***_**R**_)	0.41±0.03
**Vein density (*V***_**D**_)	5.98±1.01 m^–2^ leaf area

***Highly significant difference (*P*-value*=*<0.0001).

### Vein density: stomatal density

**Fig. 3.**
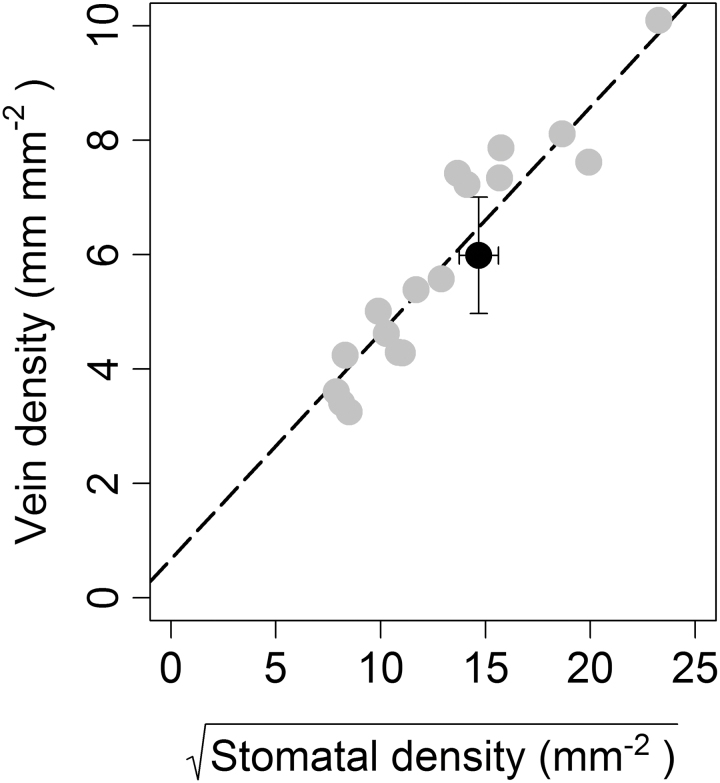
Relationship between leaf vein density and √stomatal density± SD for *H. annuus* is shown in black. Grey points are taken from Carins Murphy *et al.* (2016) and show the relationship between vein density and √stomatal density for nine hypostomatic herbaceous and woody species grown under both sun and shade conditions.

The mean vein density for *H. annuus* was 5.98 (±1.0) mm mm^–2^ (Table 1). To compare the *V*_D_:*S*_D_ of bifacial amphistomatic leaves with that of hypostomatic leaves, the *V*_D_:√*S*_D_ for *H. annuus* was overlaid with *V*_D_:√*S*_D_ data collected in a previously published study which looked at nine woody and herbaceous hypostomatic species grown in varying light conditions (Carins Murphy *et al.*, 2016) (Fig. 3); data were transformed for direct comparison with the pre-existing data. The *V*_D_:√*S*_D_ for *H. annuus* was consistent with the *V*_D_*:S*_D_ for the hypostomatic leaves (Fig. 3). If there was a construction advantage to amphistomaty that related to the simultaneous supply of water to both surfaces, we would expect that amphistomatic leaves would have a lower *V*_D_:*S*_D_ ratio than hypostomatic leaves.

### Leaf hydraulic conductance

**Fig. 4.**
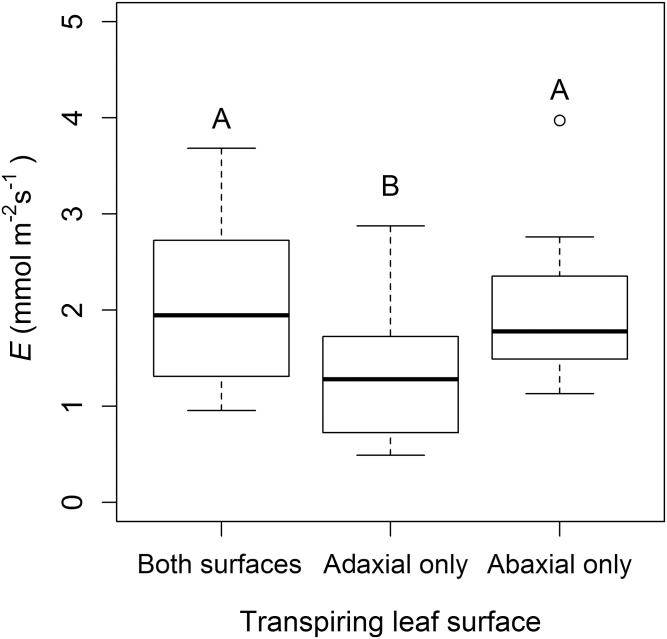
Transpiration rate (*E*) for *H. annuus* leaves connected to the flow meter under laboratory conditions. Different letters above box plots denote significant differences (*P* <0.05) in *E* between transpiring leaf surfaces.

To establish whether the stomata of both surfaces of amphistomatic leaves were opening while the leaves were attached to the flowmeter, we analysed whether the transpiration rate (*E*) for amphistomatic leaves was higher than that of functionally hypo- or epistomatic leaves (Fig. 4). The mean *E* recorded for *H. annuus* leaves during hydraulic measurements varied with transpiring leaf surface, with amphistomatic leaves highest at 2.1 mmol m^–2^ s^–1^, although not significantly higher than functionally hypostomatic leaves (mean=0.97 mmol m^–2^ s^–1^, *P*-value >0.89). Functionally epistomatic leaves, transpiring through the adaxial leaf surface only, had a significantly lower *E* than both amphistomatic and functionally hypostomatic leaves (mean=1.35 mmol m^–2^ s^–1^, *P*-value <0.05). The *E* of amphistomatic leaves (mean=2.08 mmol m^–2^ s^–1^) was not equal to the sum of epistomatic and hypostomatic *E* values (3.32 mmol m^–2^ s^–1^) which suggests that when the leaf is allowed to transpire through both surfaces under laboratory conditions, *E* through one or both surfaces is limited. 

**Fig. 5.**
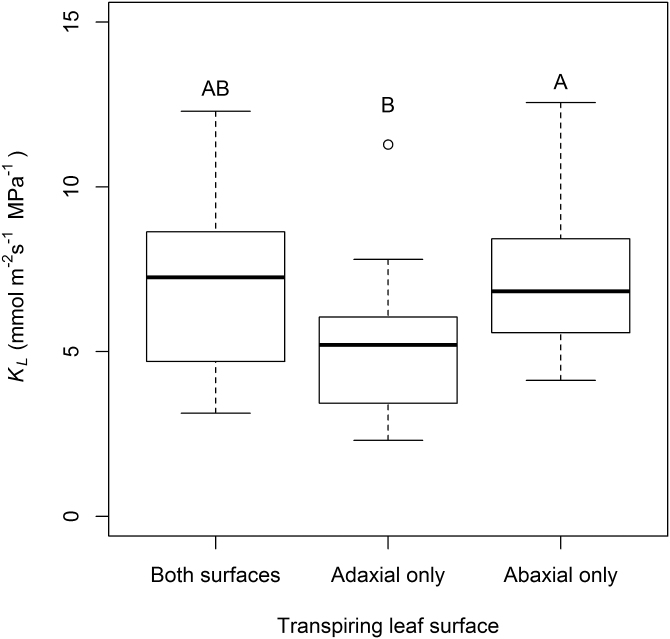
Leaf hydraulic conductance of *H. annuus* when leaves were functionally epistomatic (transpiring through the adaxial leaf surface only) and functionally hypostomatic (transpiring through the abaxial leaf surface only). Different letters above box plots denote significant differences (*P <*0.05) in *K*_leaf_ between transpiring leaf surface groups.

The mean leaf hydraulic conductance (*K*_leaf_) for amphistomatic *H. annuus* leaves (transpiring through both surfaces) was 6.9 mmol m^–2^ s^–1^ MPa^–1^ ±2.5 (SD). Compared with leaves transpiring through both surfaces, those transpiring through their abaxial surface only exhibited a slightly but not significantly higher mean *K*_leaf_ of 7.3 mmol m^–2^ s^–1^ MPa^–1^ ±2.3, which was significantly higher (*P*<0.0001) than leaves transpiring through their adaxial surface only (5.3 mmol m^–2^ s^–1^ MPa^–1^±2.3) (Fig. 5).

### Gas exchange

**Fig. 6.**
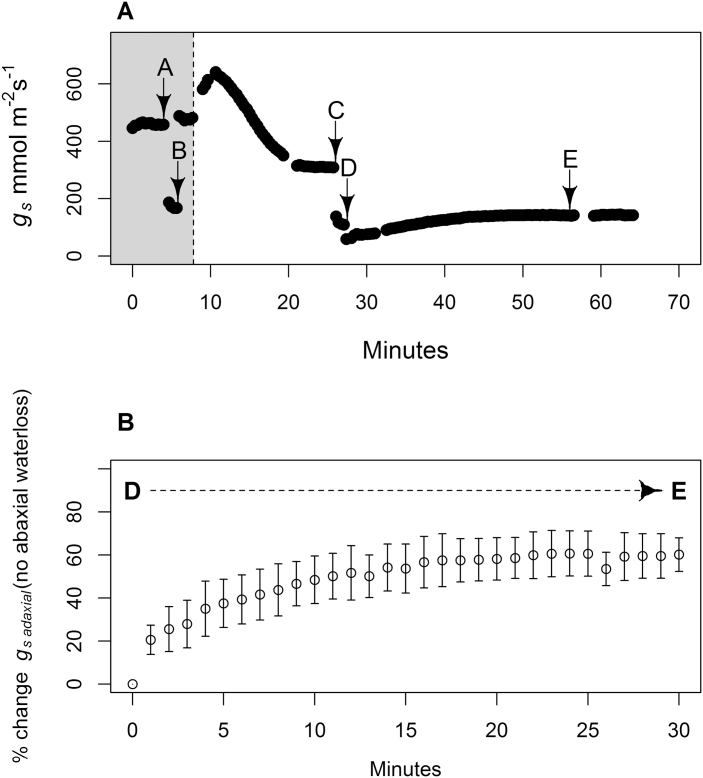
(A) A typical gas exchange trace showing stomatal conductance for *H. annuus* where ‘A’ is a stable value for both leaf surfaces transpiring, and ‘B’ is the instantaneous value for *g*_s adaxial_ only under low VPD conditions (grey area preceding the dashed line). The dashed line indicates the change from low VPD conditions (<1.2 kPa) to high VPD conditions (>2.2 kPa). ‘C’ is a stable reading for both leaf surfaces transpiring, and ‘D’ is the instantaneous value for the *g*_s adaxial_. (B) Percentage increase in adaxial *g*_*s*_ between points ‘D’ and ‘E’ which correspond to panel (A) and show high VPD conditions where water loss through the abaxial leaf surface has ceased due to preventing airflow across the abaxial leaf surface leading to humidification of surrounding air. Points are the mean of five leaves and error bars show the SD.

The length of time taken for the lower chamber to humidify once the clamps were applied, blocking airflow through the lower leaf chamber, based on the initial values for abaxial transpiration rate and relative humidity of the air at point ‘C’ (Fig. 6a) was calculated for each leaf. The calculated average time to 50% saturation with water vapour was 2.7 (±0.6) min across the five leaves, time to 75% saturation was 6.7 (±1.3) min, and the time to 95% saturation was 16.0 (±3.0) min. These calculated times are considered to be conservative as they assume that abaxial *g*_s_ and temperature remained constant. However, if the abaxial stomata opened (i.e. abaxial *g*_s_ increased), which is possible due to decreasing CO_2_ within the lower chamber, the time to saturation would be reduced.

Stomatal conductances for abaxial and adaxial surfaces of five leaves responded consistently to perturbations in VPD. Under low VPD conditions, stomata on both the adaxial and abaxial leaf surfaces were open (points ‘A’ and ‘B’, Fig. 6; Table 2) with adaxial stomata contributing just under half (average of 42%) of *g*_s total_ (Table 2). Following the transition to high VPD, the mean *g*_s total_ decreased by 52% (Table 2). The decrease in *g*_s total_ was due to stomatal closure on both leaf surfaces as the adaxial stomata under high VPD (point ‘D’, Fig. 6; Table 2) still contributed just under half (average of 45%) of the *g*_s total_ (point ‘C’, Fig. 6; Table 2).

**Table 2.** Mean gas exchange values ±SD from five *H. annuus* Ieaves

Labelled measurement points (as shown in Fig. 6)	VPD	Leaf surfaces measured	*g*_s_ (mmol m^–2^ s^–1^)	*A* (µmol m^–2^ m^–1^)	*C*_i_ (ppm)	*E* (mmol m^–2^ s^–1^)	Ψ _leaf_ (–MPa)
**A**	Low	Both	496.4±85.4	22.6±1.7	302.3±20.3	5.3±0.8	0.77±0.1
**B**	Low	Adaxial	206.9±43.1	11.9±1.9	293.8±20.6	2.9±0.5	–
**C**	High	Both	259.0±36.6	18.5±1.4	267.8±12.8	6.5±0.7	0.93±0.1
**D**	High	Adaxial	117.4±20.9	9.9±2.3	256.8±12.3	3.4±0.6	–
**E**	High	Adaxial	159.4±22.8	11.9±1.3	265.0±12.3	3.8±0.7	0.73±0.1

Ψ _leaf_ was calculated as *E*/*K*_leaf_ for ‘A’ and ‘C’ using a *K*_leaf_ of 6.9 and for ‘E’, a *K*_leaf_ of 5.3 (mmol m^–2^ s^–1^ MPa^–1^) based on whether transpiration was occurring through both leaf surfaces, or through the adaxial surface only.

The intercellular CO_2_ mole fraction (*C*_i_) was also measured at points A–E (Fig. 6; Table 2). The *C*_i_ decreased significantly (*P=*0.05) following the VPD change from low to high (points ‘A’ and ‘C’ when both leaf surfaces were transpiring (Fig. 6; Table 2), which follows the decrease in *g*_s_ induced by the increased VPD. The mean *C*_i_ of the five *H. annuus* leaves measured increased between points ‘D’ and ‘E’ (Table 2) although the increase was not significant (*P=*0.15).

If the stomatal behaviour on individual leaf surfaces was independent, we would have expected no increase in g_s adaxial_ between points ‘D’ and ‘E’ (Fig. 6) given that VPD conditions on the adaxial surface were held constant. Instead, mean *g*_s adaxial_ increased significantly between the cessation of abaxial water loss (point ‘D’ to point ‘E’, Fig. 6) (*P*<0.001).

The Ψ _leaf_ was calculated at points ‘A’,‘C’, and ‘E’ (Table 2). At points‘A’ and ‘C’ the calculation was made using the *K*_leaf_ of 6.9 mmol m^–2^ s^–1^ MPa^–1^ which was the mean *K*_leaf_ for *H. annuus* when both leaf surfaces were transpiring. For point ‘E’ Ψ _leaf_ was calculated using the *K*_leaf_ of 5.3 mmol m^–2^ s^–1^ MPa^–1^, thus representing the mean *K*_leaf_ for *H. annuus* when all transpiration was occurring through the adaxial leaf surface only. At point ‘A’ (low VPD, with both surfaces transpiring) the mean Ψ _leaf_ was –0.77 MPa. Following the increase in VPD, and associated decrease in *g*_s_, *E* for both surfaces combined was significantly higher at point ‘C’ than it was under low VPD at point ‘A’ (*P*=0.034) with a mean calculated Ψ _leaf_ of –0.93 MPa. At point ‘E’, when VPD remained high but water loss through the abaxial surface had ceased and the adaxial *g*_s_ and *E* had stabilized, the mean calculated Ψ _leaf_ of –0.73 MPa was not significantly different from the mean Ψ _leaf_ at point ‘A’ when both leaf surfaces were transpiring (*P*=0.27) (Table 2).

## Discussion

### Differences in K_*leaf*_ reflect leaf asymmetries

Greater hydraulic resistance was found in the hydraulic path supplying adaxial transpiration compared with abaxial transpiration in a typical herbaceous amphistomatic leaf. As water status can influence stomatal density (Xu and Zhou, 2008), this asymmetry in *K*_leaf_ offers a possible explanation for the asymmetry in stomatal density commonly observed in amphistomatic leaves that have greater densities of stomata allocated to the leaf abaxial surface than the adaxial surface. This aligns with the observation in previous studies that adaxial stomata are more sensitive to water stress than abaxial stomata (Aston, 1978; Mott and O’Leary, 1984), and supports the idea proposed by Milla *et al.* (2013) that increases in adaxial stomata are more likely when there is a stable water supply.

In addition to the adaxial surface possessing fewer stomata than the abaxial surface, the main anatomical asymmetries observed were in the arrangement of palisade and spongy mesophyll cells, and a significantly smaller mesophyll hydraulic path length to abaxial compared with adaxial surfaces. The differences in *K*_leaf abaxial_ and *K*_leaf adaxial_ are potentially due to the differences in the length of the hydraulic pathway through the apoplast (here we use distance from the minor veins to the inside of the epidermis as a proxy) which has been shown previously to strongly influence *K*_leaf_ in a range of plant species (Brodribb *et al.*, 2010). However, in addition to the distance from the minor veins to the epidermis, it is also possible that the hydraulic resistance is greater within the palisade mesophyll than the spongy mesophyll. While the balance between liquid and vapour flow within leaves during transpiration is not well understood and is highly temperature dependent (Buckley *et al.*, 2015, 2017), it is expected that the spongy mesophyll cells with extensive airspaces probably transport water vapour more efficiently than liquid and the tightly packed palisade cells are more likely to favour liquid transport than the spongy mesophyll (Rockwell *et al.*, 2014). In addition to potential differences in mesophyll conductance and consequences for the internal transfer of CO_2_, this may also contribute to the lower *K*_leaf_ for the adaxial leaf surface due to a higher flux of liquid as opposed to water vapour through the tissue, thus incurring greater friction.

### Stomatal traits and vein density

If there was a construction advantage to amphistomaty that related to the simultaneous supply of water to both surfaces, we would expect that amphistomatic leaves would have a lower *V*_D_:*S*_D_ ratio than hypostomatic leaves. We found no evidence that amphistomaty provides *H. annuus* with an advantage over hypostomatic species by allowing *H. annuus* to have a lower *V*_D_:*S*_D_. In fact, contrary to the idea that amphistomaty may be an efficient way to supply a greater number of stomata with water, the *V*_D_*:S*_D_ ratio was consistent with the ratios recorded from a previous study of nine hypostomatic plant species grown in both sun and shade conditions (Carins Murphy *et al.*, 2016), suggesting that any increases in stomatal density associated with amphistomaty are reflected in additional investment in the vascular network. This is also consistent with the relationship between vein length and stomatal density per unit area for both dorsiventral and isobilateral amphistomatic leaves described by Drake *et al*. (2019).

Stomatal ratios were close to those recorded in a previous study (*S*_R_*=*0.43) (Wang *et al.*, 2008). Had the *S*_D_ on the abaxial leaf surface been equal to, rather than greater than the *S*_D_ on the adaxial surface, the asymmetry in *K*_leaf_ between the leaf surfaces would have suggested an overinvestment in adaxial stomata as the lower *K*_leaf_ to the upper leaf surface would result in a greater water potential gradient through the upper leaf, which would lead to closure of adaxial stomata under conditions where abaxial stomata were able to continue to remain open. Instead, the *S*_D adaxial_ was 70.5% of the *S*_D abaxial_ which correlates remarkably well with the mean *K*_leaf adaxial_ at 73% of the *K*_leaf abaxial_.

### Changes to bulk leaf water potential show hydraulic connectivity in stomatal behaviour between leaf surfaces

When water supply was limited under conditions of high evaporative demand, we found a clear connection in the operation of stomata located on the two leaf surfaces for *H. annuus*. This is contrary to the independent stomatal behaviour observed previously between leaf surfaces of *Vicia faba* and *Xanthium strumarium* (Mott, 2007; Mott and Peak, 2018). Previous work looking at the independent behaviour of a ‘non-target’ leaf surface to changing conditions on the opposite or ‘target’ leaf surface used a decrease in humidity on the target surface and observed responses on the target and non-target surfaces. Here our approach differed in that we first ensured that stomata were operating at a high VPD where *g*_s_ was below its maximum due to lower leaf water potential (Cardoso *et al.*, 2018), prior to increasing leaf water potential by reducing the transpiration through one surface. This allowed more of the limited water available to the leaf to be directed to the opposite leaf surface.

Given that water potential can control stomatal aperture, we predicted that a decrease in *g*_s_ driven by increased evaporative demand should lead to lower *C*_i_. The results supported this, with a significant drop in *C*_i_ with the stomatal closure following the transition from low to high VPD (Table 2). However, while the increase in adaxial *g*_s_ with the reopening of the adaxial stomata after abaxial transpiration ceased was statistically significant, the increase in *C*_i_ associated with this change was not significant. This suggests that while the opposing drivers of CO_2_ uptake and water loss can both control stomatal aperture, the VPD-driven closure and subsequent re-opening of adaxial stomata observed were primarily driven by water loss and availability.

The calculated Ψ _leaf_ (which was based on *E* and the mean *K*_leaf_) indicates that the change in *E* driven by the transition from low to high VPD was sufficient to affect the bulk Ψ _leaf_ (Table 2). The change in *E* when transpiration through the abaxial surface was reduced by blocking airflow to the lower chamber of the IRGA also had a significant effect on bulk Ψ _leaf_. The results of this study suggest that for bifacial, horizontal amphistomatic leaves such as *H. annuus*, the stomata on the two leaf surfaces respond to changing conditions on the opposite leaf surface via changes in the leaf water balance driven by transpiration. This was supported by the calculations of conservation in bulk Ψ _leaf_ for the leaves prior to experiencing water stress and following the reopening of adaxial stomata when water stress was alleviated.

### Leaf hydraulics and amphistomaty

Explanations as to the selective advantages of amphistomaty are varied and include amphistomaty as a way of increasing mesophyll conductance to CO_2_ for thick leaves (Parkhurst, 1978; Mott and Michaelson, 1991). Other studies consider that fast growth and herbaceousness (Muir, 2015) are associated with the potential for greater stomatal numbers afforded by amphistomaty. However, it is important to consider how water moves through amphistomatic leaves and the stomatal response of the two surfaces. Leaves which are orientated horizontally and receive direct irradiance and the associated heat and evaporative load on their adaxial surface should theoretically experience the greatest water potential gradient within the upper portion of the leaf (Richardson *et al.*, 2017). Compounding this effect in *H. annuus* is the fact that the upper surface of the leaf has a lower hydraulic conductance than the lower surface. These features would probably cause preferential closure of adaxial stomata under evaporative load, leading to underutilization of the adaxial stomata. However, the proportionally lower density of stomata on the adaxial leaf surface would decrease the potential overinvestment in stomata associated with this hydraulic asymmetry.

Overall, we did not identify any hydraulic advantage when the leaves were functionally amphistomatic as opposed to functionally hypostomatic or epistomatic, because stomata on both surfaces are limited by their dependence on the capacity of the vascular system to supply water. We did, however, identify other hydraulic implications of amphistomatic stomatal arrangement on internal water flow through the bifacial leaf. Thus, asymmetrical hydraulic conductances between leaf surfaces of strongly bifacial amphistomatic leaves suggests that amphistomatic leaves with an even stomatal distribution (*S*_R_=0.5) may be at a disadvantage compared with leaves with more abaxial than adaxial stomata. This disadvantage may explain the uneven stomatal ratio observed in *Helianthus* as well as additional species from a range of families (Muir, 2015). Additionally, our results show significant coordination between stomata on both surfaces. While the water potential gradient outside the xylem allows for a degree of independence in stomatal behaviour between leaf surfaces, we identified a level of coordination which is consistent with hydraulic models, suggesting that changes in bulk leaf water potential should impact the gas exchange for the entire leaf.

## References

[CIT0001] Aston M 1978 Differences in the behaviour of adaxial and abaxial stomata of amphistomatous sunflower leaves: inherent or environmental? Functional Plant Biology 5, 211–218.

[CIT0002] Beerling DJ, Kelly CK 1996 Evolutionary comparative analyses of the relationship between leaf structure and function. New Phytologist 134, 35.

[CIT0003] Brodribb TJ, Feild TS, Jordan GJ 2007 Leaf maximum photosynthetic rate and venation are linked by hydraulics. Plant Physiology 144, 1890–1898. 17556506 10.1104/pp.107.101352 PMC1949879

[CIT0004] Brodribb TJ, Feild TS, Sack L 2010 Viewing leaf structure and evolution from a hydraulic perspective. Functional Plant Biology 37, 488–498.

[CIT0005] Brodribb TJ, Holbrook NM 2006 Declining hydraulic efficiency as transpiring leaves desiccate: two types of response. Plant, Cell & Environment 29, 2205–2215. 10.1111/j.1365-3040.2006.01594.x 17081253

[CIT0006] Buckley TN, John GP, Scoffoni C, Sack L 2015 How does leaf anatomy influence water transport outside the xylem? Plant Physiology 168, 1616–1635. 26084922 10.1104/pp.15.00731 PMC4528767

[CIT0007] Buckley TN, John GP, Scoffoni C, Sack L 2017 The sites of evaporation within leaves. Plant Physiology 173, 1763–1782. 28153921 10.1104/pp.16.01605 PMC5338672

[CIT0008] Cardoso AA, Brodribb TJ, Lucani CJ, DaMatta FM, McAdam SAM 2018 Coordinated plasticity maintains hydraulic safety in sunflower leaves. Plant, Cell & Environment 41, 2567–2576. 10.1111/pce.13335 29748980

[CIT0009] Carins Murphy MR, Jordan GJ, Brodribb TJ 2012 Differential leaf expansion can enable hydraulic acclimation to sun and shade. Plant, Cell & Environment 35, 1407–1418. 10.1111/j.1365-3040.2012.02498.x 22339445

[CIT0010] Carins Murphy MR, Jordan GJ, Brodribb TJ 2014 Acclimation to humidity modifies the link between leaf size and the density of veins and stomata. Plant, Cell & Environment 37, 124–131. 10.1111/pce.12136 23682831

[CIT0011] Carins Murphy MR, Jordan GJ, Brodribb TJ 2016 Cell expansion not cell differentiation predominantly co-ordinates veins and stomata within and among herbs and woody angiosperms grown under sun and shade. Annals of Botany 118, 1127–1138. 27578763 10.1093/aob/mcw167 PMC5963197

[CIT0012] Cochard H, Nardini A, Coll L 2004 Hydraulic architecture of leaf blades: where is the main resistance? Plant, Cell & Environment 27, 1257–1267.

[CIT0013] Damour G, Simonneau T, Cochard H, Urban L 2010 An overview of models of stomatal conductance at the leaf level. Plant, Cell & Environment 33, 1419–1438. 10.1111/j.1365-3040.2010.02181.x 20545879

[CIT0014] Drake PL, de Boer HJ, Schymanski SJ, Veneklaas EJ 2019 Two sides to every leaf: water and CO_2_ transport in hypostomatous and amphistomatous leaves. New Phytologist 222, 1179–1187. 30570766 10.1111/nph.15652

[CIT0015] Jordan GJ, Carpenter RJ, Brodribb TJ 2014 Using fossil leaves as evidence for open vegetation. Palaeogeography, Palaeoclimatology, Palaeoecology 395, 168–175.

[CIT0016] Korson L, Drost-Hansen W, Millero FJ 1969 Viscosity of water at various temperatures. Journal of Physical Chemistry 73, 34–39.

[CIT0017] McAdam SA, Brodribb TJ 2016 Linking turgor with ABA biosynthesis: implications for stomatal responses to vapor pressure deficit across land plants. Plant Physiology 171, 2008–2016. 27208264 10.1104/pp.16.00380 PMC4936570

[CIT0018] McAdam SAM, Brodribb TJ 2018 Mesophyll cells are the main site of abscisic acid biosynthesis in water-stressed leaves. Plant Physiology 177, 911–917. 29735726 10.1104/pp.17.01829 PMC6052997

[CIT0019] McKown AD, Guy RD, Quamme L, Klápště J, La Mantia J, Constabel CP, El-Kassaby YA, Hamelin RC, Zifkin M, Azam MS 2014 Association genetics, geography and ecophysiology link stomatal patterning in *Populus trichocarpa* with carbon gain and disease resistance trade-offs. Molecular Ecology 23, 5771–5790. 25319679 10.1111/mec.12969

[CIT0020] Milla R, de Diego-Vico N, Martín-Robles N 2013 Shifts in stomatal traits following the domestication of plant species. Journal of Experimental Botany 64, 3137–3146. 23918960 10.1093/jxb/ert147

[CIT0021] Mott KA 2007 Leaf hydraulic conductivity and stomatal responses to humidity in amphistomatous leaves. Plant, Cell & Environment 30, 1444–1449. 10.1111/j.1365-3040.2007.01720.x 17897414

[CIT0022] Mott KA, Cardon ZG, Berry JA 1993 Asymmetric patchy stomatal closure for the two surfaces of *Xanthium strumarium* L. leaves at low humidity. Plant, Cell & Environment 16, 25–34.

[CIT0023] Mott KA, Michaelson O 1991 Amphistomy as an adaptation to high light intensity in *Ambrosia cordifolia* (Compositae). American Journal of Botany 78, 76–79.

[CIT0024] Mott KA, O’Leary JW 1984 Stomatal behavior and CO_2_ exchange characteristics in amphistomatous leaves. Plant Physiology 74, 47–51. 16663384 10.1104/pp.74.1.47 PMC1066622

[CIT0025] Mott KA, Parkhurst DF 1991 Stomatal responses to humidity in air and Helox. Plant, Cell & Environment 14, 509–515.

[CIT0026] Mott KA, Peak D 2018 Effects of the mesophyll on stomatal responses in amphistomatous leaves. Plant, Cell & Environment 41, 2835–2843. 10.1111/pce.13411 30073677

[CIT0027] Muir CD 2015 Making pore choices: repeated regime shifts in stomatal ratio. Proceedings of the Royal Society: B Biological Sciences 282, 20151498. 10.1098/rspb.2015.1498 PMC4632635 26269502

[CIT0028] Muir CD 2018 Light and growth form interact to shape stomatal ratio among British angiosperms. New Phytologist 218, 242–252. 29288622 10.1111/nph.14956

[CIT0029] Parkhurst DF 1978 The adaptive significance of stomatal occurrence on one or both surfaces of leaves. Journal of Ecology 66, 367–383.

[CIT0030] Parkhurst DF 1994 Tansley review no. 65. Diffusion of CO_2_ and other gases inside leaves. New Phytologist 126, 449–479. 10.1111/j.1469-8137.1994.tb04244.x 33874469

[CIT0031] R Core Team 2014 R: a language and environment for statistical computing. Vienna, Austria: R Foundation for Statistical Computing.

[CIT0032] Richardson F, Brodribb TJ, Jordan GJ 2017 Amphistomatic leaf surfaces independently regulate gas exchange in response to variations in evaporative demand. Tree Physiology 37, 869–878. 28898992 10.1093/treephys/tpx073

[CIT0033] Rockwell FE, Holbrook NM, Stroock AD 2014 The competition between liquid and vapor transport in transpiring leaves. Plant Physiology 164, 1741–1758. 24572172 10.1104/pp.114.236323 PMC3982738

[CIT0034] Sack L, Holbrook NM 2006 Leaf hydraulics. Annual Review of Plant Biology 57, 361–381. 10.1146/annurev.arplant.56.032604.144141 16669766

[CIT0035] Sack L, Melcher PJ, Zwieniecki MA, Holbrook NM 2002 The hydraulic conductance of the angiosperm leaf lamina: a comparison of three measurement methods. Journal of Experimental Botany 53, 2177–2184. 12379784 10.1093/jxb/erf069

[CIT0036] Sack L, Streeter CM, Holbrook NM 2004 Hydraulic analysis of water flow through leaves of sugar maple and red oak. Plant Physiology 134, 1824–1833. 15064368 10.1104/pp.103.031203 PMC419854

[CIT0037] Wang Y, Noguchi K, Terashima I 2008 Distinct light responses of the adaxial and abaxial stomata in intact leaves of *Helianthus annuus* L. Plant, Cell & Environment 31, 1307–1316. 10.1111/j.1365-3040.2008.01843.x 18537998

[CIT0038] Xu Z, Zhou G 2008 Responses of leaf stomatal density to water status and its relationship with photosynthesis in a grass. Journal of Experimental Botany 59, 3317–3325. 18648104 10.1093/jxb/ern185 PMC2529243

[CIT0039] Zwieniecki MA, Brodribb TJ, Holbrook NM 2007 Hydraulic design of leaves: insights from rehydration kinetics. Plant, Cell & Environment 30, 910–921. 10.1111/j.1365-3040.2007.001681.x 17617819

